# Iranian terrestrial isopods of the family Cylisticidae Verhoeff, 1949 with a description of a new species (Isopoda, Oniscidea)

**DOI:** 10.3897/zookeys.582.7199

**Published:** 2016-04-21

**Authors:** Ghasem M. Kashani

**Affiliations:** 1 Department of Biology, Faculty of Sciences, University of Zanjan, Zanjan, Iran

**Keywords:** Oniscidea, Cylisticidae, Cylisticus, Cylisticoides, new species, Iran

## Abstract

In the present study, the terrestrial isopods of the family Cylisticidae in Iran are investigated. Geographical distributions of two formerly reported species from Iran, namely *Cylisticoides angulatus* Schmalfuss, 2003 and *Cylisticus rotundifrons* (Schmalfuss, 1986), are expanded. *Cylisticus masalicus* **sp. n**. is described and its diagnostic characters are figured.

## Introduction

Verhoeff (1949) established the subfamily Cylisticinae for the genus *Cylisticus* Schnitzler, 1853 in the family “Porcellionidae”. Strouhal (1953) did not accept the recognition of Cylisticinae as a separate group and proposed the inclusion of all forms with five pairs of lungs in the subfamily Trachelipinae. Vandel (1962) followed Verhoeff’s view and actually raised Cylisticidae to family level while Schmalfuss (2003a) questioned the validity of the family.

Members of Cylisticidae are characterized by: strongly convex body; exoantennal conglobation ability, though the head and telson are poorly adapted to conglobation; pereon tergite I without modifications or with grooves to receive the antennae; head with developed lateral and median lobes; supra-antennal line absent; antenna long; flagellum with two articles; pleopod exopodites I-V with wrinkled, partly covered, “*Trachelipus*-type”, or with internal, covered, “*Porcellio*-type” lungs (Schmalfuss 1992, 2003a; Schmidt 2003). There are no clear synapomorphies defining the taxon.

Cylisticidae species are distributed from central Europe to central Asia. According to the world catalogue of terrestrial isopods (Schmalfuss 2003b), the family comprises five genera, namely *Cylisticoides* Schmalfuss, 2003, *Cylisticus* Schnitzler, 1853, *Lepinisticus* Manicastri & Taiti, 1983, *Parcylisticus* Verhoeff, 1943, and *Troglocylisticus* Ferrara & Taiti, 1983. Schmalfuss (2003b) included *Cylisticoides* in the family Cylisticidae with a question mark, since he believed that the superficial similarity of the genus *Cylisticoides* with the other genera might be due to convergence.

Schmalfuss (1986) recorded the genus *Cylisticus* from Iran, based on three female specimens, as *Cylisticus* sp. I. In the same contribution, he also described *Cylisticus rotundifrons* which was later (Schmalfuss 2003a) transferred to the new genus *Cylisticoides*. He also named another female specimen as *Cylisticus* sp. II, which was later described as *Cylisticoides angulatus* (Schmalfuss 2003a).

In the present study, new records for *Cylisticoides angulatus* and *Cylisticus rotundifrons* in Iran are presented and *Cylisticus masalicus* sp. n. is described.

## Material and methods

**Figure 1.**
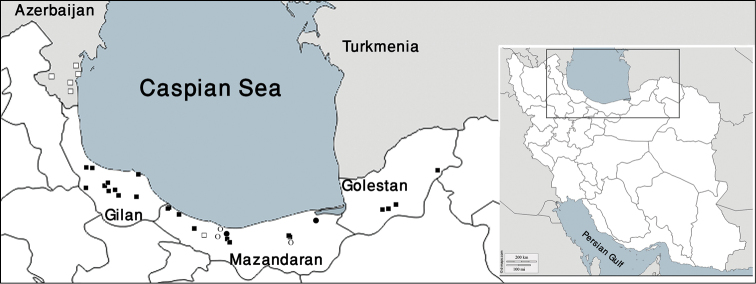
Map of Iran with the northern part enlarged, indicating the sampling localities of *Cylisticoides angulatus* (□ previous records, ■ new records) and *Cylisticus rotundifrons* (○ previous records, ● new records).

The material examined herein was collected in Iran (Fig. 1), mostly from May 2014 to September 2015. The specimens were collected by hand and preserved in 96% ethanol. Drawings were made using a drawing tube on a Nikon Y-IDT compound microscope. Type material of the newly described species is deposited in the Zoological Museum, University of Tehran (ZUTC), the Iranian Research Institute of Plant Protection (IRIPP) and in the personal collection of the author (PCGMK).

## Taxonomy

### Order Isopoda Latreille, 1817 Suborder Oniscidea Latreille, 1802 Family Cylisticidae Verhoeff, 1949

#### Cylisticoides rotundifrons

Taxon classificationAnimaliaIsopodaCylisticidae

(Schmalfuss, 1986)

Cylisticus rotundifrons Schmalfuss, 1986: p. 394; Schmalfuss 2003b: 99.

##### Material examined.

Amol to Chamestan, Belvich village, 36°28.3'N, 52°10.0'E, elev. 60 m, 29 July 2014, leg. G.M. Kashani, two females (PCGMK 1960); Neka to Behshahr, Pasandrez forests, 36°40.0'N, 53°36.7'E, elev. 300 m, 31 July 2014, leg. G.M. Kashani, one female (PCGMK 1998).

##### Distribution.

N Iran.

##### Remarks.

Schmalfuss (1986) described *Cylisticus rotundifrons* from a female specimen collected at 12 km NE Zirab, Iran. He also named two females from Noor district as *Cylisticus* aff. *rotundifrons*. Schmalfuss (2003a) transferred the species to the genus *Cylisticoides*. In the present study, no male specimen was found either. Despite been relatively widely distributed in northern Iran (Fig. 1), *Cylisticus rotundifrons* does not seem to be common. Though no male is known for the species, it can be readily distinguished from *Cylisticoides angulatus*, the only congeneric species, from the rounded (vs. angled) postero-lateral margin of pereon-tergite I and the lack of a ridge on lateral margin of pereon-tergite I (present in *Cylisticoides angulatus*). Based on current knowledge, *Cylisticus rotundifrons* is restricted to northern Iran.

#### Cylisticoides angulatus

Taxon classificationAnimaliaIsopodaCylisticidae

Schmalfuss, 2003

##### Material examined.

Gorgan, Naharkhoran district, 36°46.8'N, 54°27.8'E, elev. 430 m, 1 August 2014, leg. G.M. Kashani, eight males, eight females and four juv. (PCGMK 2010); 15 km S Jalin, 36°42.5'N, 54°35.3'E, elev. 900 m, 1 August 2014, leg. G.M. Kashani, one male, three females and seven juv. (PCGMK 2016); Ramian to Shahrood, 36°52.1'N, 55°13.4'E, elev. 1375 m, 2 August 2014, leg. G.M. Kashani, one male, three (PCGMK 2021); Poonel to Sangdeh, 37°32.0'N, 48°56.7'E, elev. 420 m, 14 August 2014, leg. G.M. Kashani, five females and three juv. (PCGMK 1800); Poonel to Sangdeh, 3 km to Zendaneh, 37°32.2'N, 48°45.1'E, elev. 1500 m, 14 August 2014, leg. G.M. Kashani, three males, four females and two juv.. (PCGMK 1801); Fooman, Ghaleh Roodkhan, 37°04.5'N, 49°14.9'E, elev. 330 m, 15 August 2014, leg. G.M. Kashani, six males, two females and thirteen juv. (PCGMK 1807); Shaft, Siahmazgi, 37°01.3'N, 49°16.4'E, elev. 400 m, 14 August 2014, leg. G.M. Kashani, four males, four females (PCGMK 1810); Someh-Sara, 37°17.7'N, 49°18.6'E, elev. 15 m, 16 August 2014, leg. G.M. Kashani, five males, two females and three juv. (PCGMK 1821); Fooman to Roodbar, 37°10.8'N, 49°33.4'E, elev. 50 m, 17 August 2014, leg. G.M. Kashani, one female (PCGMK 1822); Saravan, 37°07.8'N, 49°38.9'E, elev. 60 m, 17 August 2014, leg. G.M. Kashani, six males and one juv. (PCGMK 1823); Kiashahr port, 37°25.6'N, 49°57.6'E, 18 August 2014, leg. G.M. Kashani, one female (PCGMK 1837); Siahkal to Deylaman, Loonak village, 37°03.4'N, 49°53.7'E, 19 August 2014, leg. G.M. Kashani, one female and three juv. (PCGMK 1842); Klardasht to Abbasabad, 36°37.5'N, 51°06.4'E, elev. 400 m, 12 September 2014, leg. G.M. Kashani, one subadult (PCGMK 1878); Tonekabon, Darbar village, 36°39.4'N, 50°47.7'E, elev. 500 m, 13 September 2014, leg. G.M. Kashani, two males and eight females (PCGMK 1885); Ramsar to Javaherdeh, 36°54.6'N, 50°36.7'E, elev. 170 m, 14 September 2014, leg. G.M. Kashani, one female (PCGMK 1891); Ramsar to Javaherdeh, 36°52.6'N, 50°33.4'E, elev. 730 m, 14 September 2014, leg. G.M. Kashani, four males, one female and eighteen juv. (PCGMK 1896); Masooleh, 19 July 2004, leg. G.M. Kashani, two females (PCGMK 1174); Galikesh to Bojnurd, Golestan National Park, 37°23.0'N, 55°50.7'E, 6 May 2008, leg. G.M. Kashani, two females (PCGMK 1180); 6 km S Shirgah, 36°15.9'N, 52°53.9'E, elev. 210 m, 8 June 2015, leg. G.M. Kashani, two females (PCGMK 2088); Shirgah, 36°16.9'N, 52°53.2'E, elev. 240 m, 8 June 2015, leg. G.M. Kashani, three males and six females (PCGMK 2090); Amol to Chamestan, Belvich village, 36°28.3'N, 52°10.0'E, elev. 60 m, 4 September 2015, leg. G.M. Kashani, twelve males and eighteen females (PCGMK 2102); 17 km S Amol, 36°16.3'N, 52°22.0'E, elev. 500 m, 4 September 2015, leg. G.M. Kashani, five males and four females (PCGMK 2104).

##### Distribution.

SE Azerbaijan; N Iran.

##### Remarks.

Schmalfuss (2003a) established a new genus and species for specimens collected from southeastern Azerbaijan, namely *Cylisticoides angulatus*. He also assigned one female specimen collected from Dashte-Nazir, Iran, previously named as *Cylisticus* sp. II (Schmalfuss 1986), to this species.

In the present study, *Cylisticoides angulatus* was collected at a broad range of localities in northern Iran (Fig. 1). The preferred habitat for this species seems to be the bark of decaying trees in old forests.

#### Cylisticus masalicus sp. n.

Taxon classificationAnimaliaIsopodaCylisticidae

http://zoobank.org/6A53EAA9-369C-4962-ABB8-E65E7B0E1DA7

##### Material examined.

Holotype: male, 11 mm, IRAN, Gilan, Masal, 37°19.0'N, 48°59.0'E, elev. 600 m, 19 March 2014, leg. G.M. Kashani (ZUTC 5786).

Paratypes: same data as holotype, one male and two females (ZUTC 5787); same data as holotype, one male and one female (IRIPP Iso-1063); same data as holotype, three males and four females (PCGMK 1749); Gachsar to Marzanabad, 5 km to Dozdband, 36°16.2'N, 51°14.6'E, 26 July 2014, leg. G.M. Kashani, one female (PCGMK 1921); Noor to Kojour, 36°26.2'N, 51°53.3'E, elev. 790 m, 28 July 2014, leg. G.M. Kashani, one female (PCGMK 1942); Noor to Kojour, Kodir village, 36°26.4'N, 51°51.6'E, elev. 1435 m, 28 July 2014, leg. G.M. Kashani, one female and one juv. (PCGMK 1946); Kojour to Galandrood, 36°26.7'N, 51°50.7'E, elev. 1480 m, 28 July 2014, leg. G.M. Kashani, one male (PCGMK 1952); Poonel to Sangdeh, 37°33.4'N, 48°41.5'E, elev. 2200 m, 14 August 2014, leg. G.M. Kashani, one male and one female (IRIPP Iso-1062); Poonel to Sangdeh, 37°33.4'N, 48°41.5'E, elev. 2200 m, 14 August 2014, leg. G.M. Kashani, four males, five females and one juv. (PCGMK 1803); 29 km to Asalem, 37°37.5'N, 48°48.6'E, elev. 2220 m, 14 August 2014, leg. G.M. Kashani, four females (PCGMK 1804); 10 km to Shaft, 37°06.2'N, 49°23.8'E, elev. 80 m, 15 August 2014, leg. G.M. Kashani, one female (PCGMK 1813); Siahkal to Deylaman, 10 km to Deylaman, 36°56.2'N, 49°51.8'E, elev. 1500 m, 19 August 2014, leg. G.M. Kashani, one male (PCGMK 1845); Boomehen to Amol, Ploor village, 35°50.9'N, 52°03.2'E, elev. 2200 m, 11 September 2014, leg. G.M. Kashani, two females (PCGMK 1846); Tonekabon, Darbar village, 36°42.7'N, 50°50.7'E, elev. 220 m, 13 September 2014, leg. G.M. Kashani, three females and two males (PCGMK 1882); Galesh-Mahalleh to Jannat-Roodbar, 36°49.3'N, 50°41.3'E, elev. 370 m, 13 September 2014, leg. G.M. Kashani, one female (PCGMK 1888); Ramsar to Javaherdeh, 36°52.5'N, 50°33.3'E, elev. 770 m, 14 September 2014, leg. G.M. Kashani, one male and one female (PCGMK 1893); Amlash, Khorma village, 37°04.5'N, 49°58.9'E, elev. 270 m, 14 September 2014, leg. G.M. Kashani, one male (PCGMK 1902); Rahim-Abad to Ziaz, 36°56.5'N, 50°16.5'E, elev. 220 m, 15 September 2014, leg. G.M. Kashani, two males and one female (PCGMK 1912).

##### Diagnosis.

Cephalon with well developed quadrangular lateral lobes; median process pointed upwards; male pereopod VII ischium subrectangular; male pleopod endopodite I with apical part slightly bent outwards, bearing some setae.

##### Description.

**Figure 2.**
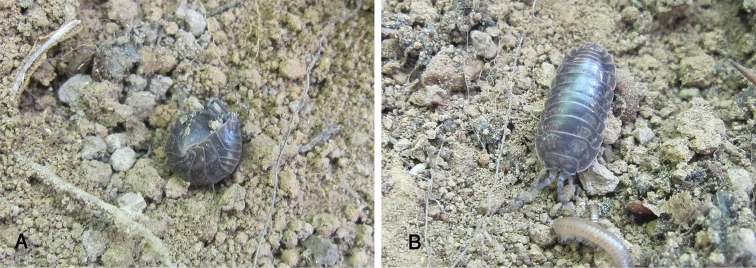
*Cylisticus masalicus* sp. n.; **A** conglobated **B** active.

**Figure 3.**
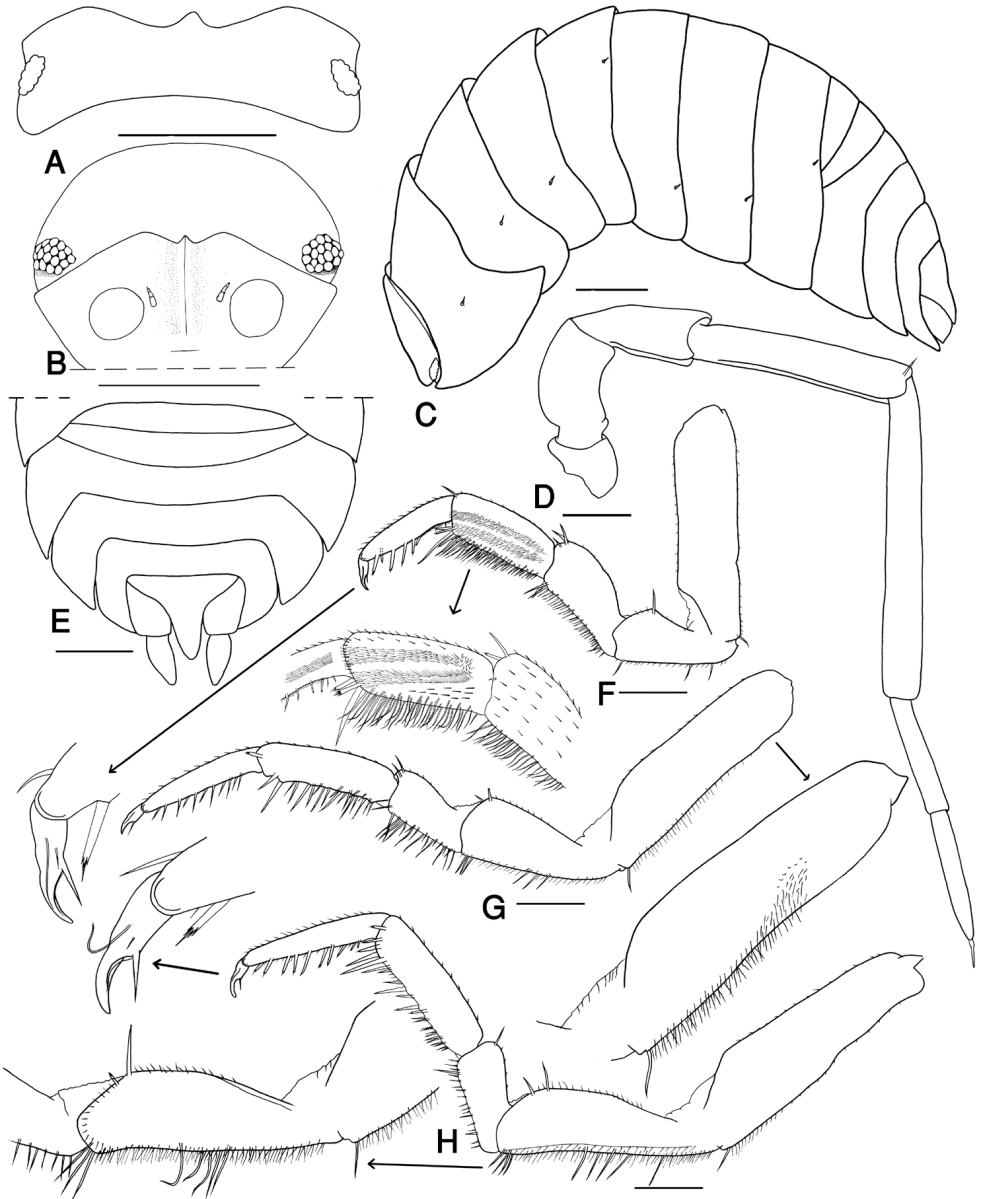
*Cylisticus masalicus* sp. n., male, paratype. **A** cephalon dorsal view **B** cephalon frontal view **C** left side of the body showing the position of noduli laterales **D** antenna **E** telson and uropods **F** pereopod I and enlarged carpus and dactylus **G** pereopod VI and enlarged basis **H** pereopod VII and enlarged ischium. Scale: 1 mm (**A–C**); 0.5 mm (**D–H**).

Maximum length of both male and female, 15 mm. Color slaty gray with the usual pale muscle spots (Fig. 2A, B). Exoantennal conglobation (Fig. 2A) and body semi-circular in cross section. Cephalon with well developed quadrangular lateral lobes; median process pointed upwards, not surpassing lateral lobes in frontal view; vertex smooth, eyes with 20 to 22 ommatidia (Fig. 3A, B). Antenna long and slender, bent on the back when conglobating; fifth article of peduncle slightly longer than flagellum, with length:width ratio 7:1; flagellum with two articles of the same size (Fig. 3D).

Pereon smooth. Pereonite I with angular concavity on posterolateral margin. Noduli laterales on pereonite IV more than twice distant from the lateral margins than those on pereonite III (Fig. 3C).

Pereopod I ischium triangular, carpus with depression on rostral surface equipped with slender scales, dactylus with one dactylar and one ungual seta (Fig. 3F).

**Figure 4.**
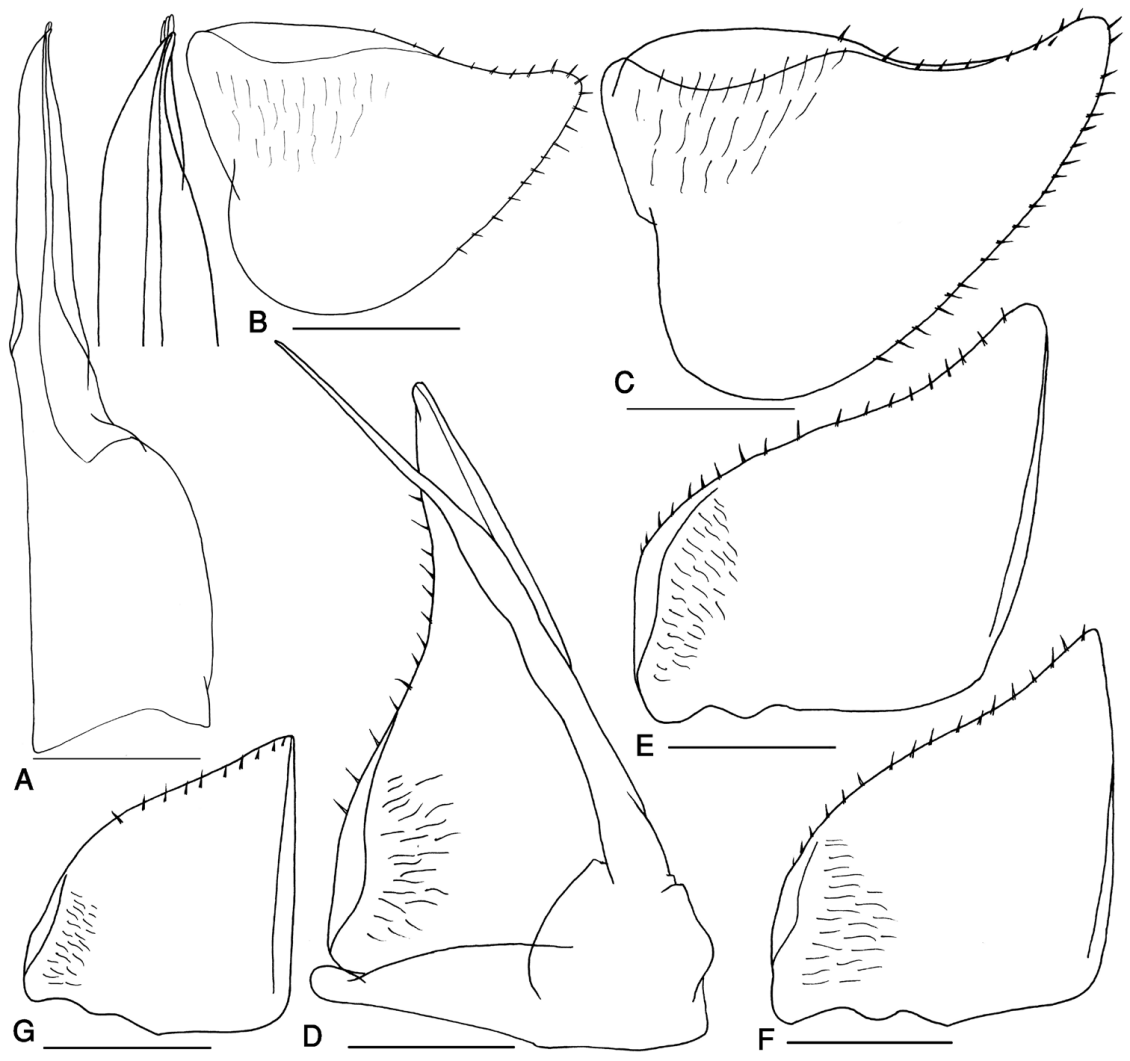
*Cylisticus masalicus* sp. n., male, paratype. **A** pleopod endopodite I **B–C** pleopod exopodite I **D** pleopod II **E** pleopod exopodite III **F** pleopod exopodite IV **G** pleopod exopodite V. Scales = 0.5 mm.

Pleon as broad as pereon (Fig. 3E). Telson triangular, with concave sides and rounded apex, surpassing uropod-protopodites. Uropod-exopodites short, 2/3 as long as telson (Fig. 3E). Pleopod exopodites I–V with polyspiracular internal lungs (Fig. 4B–F).

Male: Pereopods I–VII merus and carpus with brushes of trifid setae, less dense in posterior ones (Fig. 3F–H). Pereopod VI ischium on sternal margin with three long spiny setae medially and three long spiny setae distally (Fig. 3G), pereopod VII ischium subrectangular, ventral side with a hairy brush of small setae, medially with six and distally with four long spiny setae (Fig. 3H). Pleopod endopodite I straight with apical part slightly bent outwards equipped with some short setae (Fig. 4A); exopodite I with rounded hind lobe, outer margin with a row of small setae (Fig. 4B, C). Pleopod endopodite II longer than exopodite; exopodite triangular with a line of setae on outer margin (Fig. 4D). Pleopod exopodites III–V as in Fig. 4E–G.

##### Etymology.

The name of the species is after the type locality, the rain forests around Masal.

##### Distribution.

N Iran.

##### Remarks.

**Figure 5.**
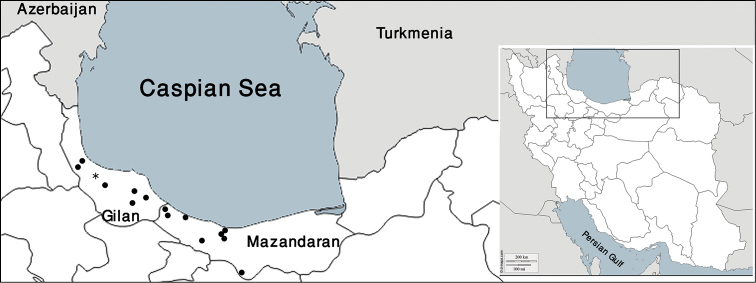
Map of Iran with the northern part enlarged, indicating the sampling localities of *Cylisticus masalicus*. * indicates the type locality.

In the examination of a collection of terrestrial isopods from northern Iran, Schmalfuss (1986) reported the genus *Cylisticus* for the first time based on three female specimens, which he cited as *Cylisticus* sp.I. This was the sole account for this genus up to now. In the present study, *Cylisticus masalicus* sp. n. is described. It has a broad distribution in the rain forests of the country (Fig. 5). According to the illustrations presented by Schmalfuss (1986) for the specimens named *Cylisticus* sp.I. and their collecting localities that lies inside the geographical range of the species (Fig. 5), those specimens presumably belong to *Cylisticus masalicus* as well.

*Cylisticus masalicus* sp. n. differs from other species of the genus by the straight apex of the male pleopod endopodite I which is also common in *Parcylisticus* species. The new species, however, is well assignable to the genus *Cylisticus* for its smooth body surface and short uropod exopodites. This species is similar to *Cylisticus birsteini* Borutzky, 1961 and *Cylisticus iners* Budde-Lund, 1880 but differs from the former in the shape of the male pleopod endopodite I, and from the latter in the position of noduli laterales and the shape of telson.

## Supplementary Material

XML Treatment for Cylisticoides rotundifrons

XML Treatment for Cylisticoides angulatus

XML Treatment for Cylisticus masalicus
